# Open questions in organic crystal polymorphism

**DOI:** 10.1038/s42004-020-00388-9

**Published:** 2020-10-19

**Authors:** Aurora J. Cruz-Cabeza, Neil Feeder, Roger J. Davey

**Affiliations:** 1 Department of Chemical Engineering and Analytical Science, The University of Manchester, Oxford Road, Manchester, M13 9PL UK; 2 Neil Feeder Consulting Ltd., 9 Betony Vale, Royston, Hertfordshire SG8 9TS UK

**Keywords:** Physical chemistry, Crystal engineering, Structure elucidation

## Abstract

Polymorphs, crystals with different structure and properties but the same molecular composition, arise from the subtle interplay between thermodynamics and kinetics during crystallisation. In this opinion piece, the authors review the latest developments in the field of polymorphism and discuss standing open questions.

Be they medicines, foodstuffs, paints or electronics, many of the products that we consume contain active crystalline components. The structure and the properties of such crystalline solids are dictated by intermolecular interactions which bring molecules together in perfectly ordered symmetric arrangements—crystal structures (Fig. 1)^1^. The regularity of these structures is manifest to the naked eye in the form crystallites of regular and symmetric shapes (also known as crystal habits and morphologies), and their exquisite internal structures can be determined at the molecular level by techniques such as X-ray diffraction.

**Fig. 1:**
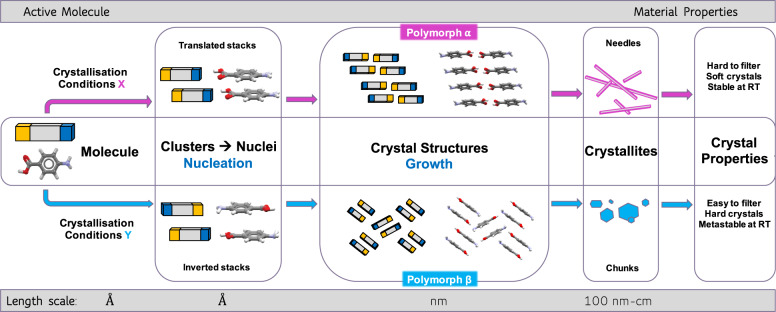
Supramolecular assembly evolution of p-aminobenzoic acid (*p*ABA) from a single molecule into crystallites of two different polymorphs. The assembly occurs via two different routes (pink and blue) as a consequence of two different types of crystallisation conditions (X and Y), which leads to different early assembly, polymorphic structure, crystal shapes and material properties^1^.

Crystallisation is the process by which molecular crystals are produced—with crystallisation from a liquid solution being the most common in industry. Two steps are involved in crystallisation: (i) nucleation, which is the process leading to the birth of a crystal nucleus and (ii) growth, which is the process by which molecules get incorporated into such nuclei, leading to macroscopic crystals. Because intermolecular interactions are soft ‘bonds’ and because molecules possess shape, the competition between maximum space filling and utilisation of strongest possible interactions can result in the nucleation and growth of different crystal structures (with identical composition and similar energies) under different crystallisation conditions (solvent, composition, purity, temperature, pressure). This phenomenon, known as crystal polymorphism (Fig. 1), was first noticed almost 200 years ago for inorganic compounds by Mitscherlich and soon after for organic compounds by the German chemists Liebig and Wöhler^2,3^. The phenomenon remained a curiosity at the time and was barely studied until, in the late 1960s, its importance to the development of drugs and other products began to be realised. Because each polymorph of a given molecule has a unique crystal structure, it would also have its own unique physical properties (such as mechanical hardness, solubility, colour, melting point etc). To manufacture and deliver crystalline based consumer products which perform consistently, therefore, it is vital to control and deliver the same polymorphic form across each batch of crystals (Fig. 1). In this context, what have we learnt and what are the open questions in organic crystal polymorphism?

## Molecular basis of polymorphism

### Q1. Why are some compounds polymorphic and others not?

**Fig. 2:**
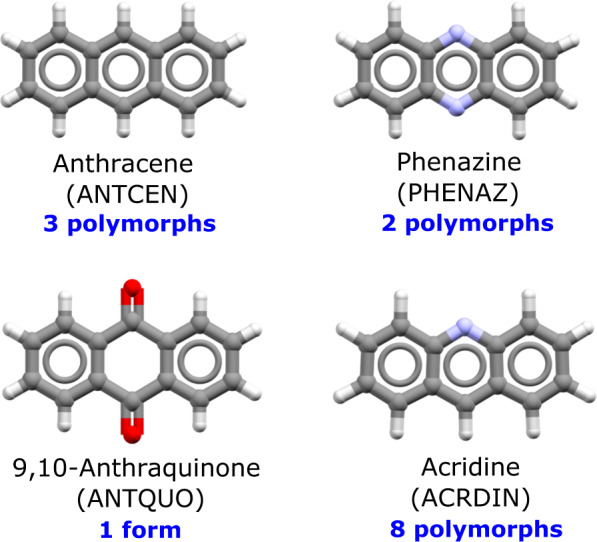
Similar compounds can behave very differently when it comes to polymorphism. Molecular structure, Cambridge Structural Database six-letter family refcode and number of polymorphs for anthracene, phenazine, 9,10-anthraquinone and acridine.

This is perhaps the first fundamental question which remains open. In some cases, compounds having very similar chemical structure behave in a completely different manner when it comes to polymorphism. To our knowledge, 9,10-anthraquinone crystallises in one unique crystal form, phenazine crystallises in two polymorphs, anthracene in three polymorphs, whilst acridine has been reported to have at least nine polymorphs (Fig. 2)^4^. We know from various databases that between 37–66% of compounds exhibit polymorphism^3^. The vast majority of polymorphic compounds are reported to only have a pair of polymorphs (89%), 9% three polymorphs and only 2% four polymorphs or more^2^. Yet, it remains impossible to know, based on molecular structure alone, whether a compound will be polymorphic or indeed how to decide if all possible polymorphs have been found in experimentation.

### Q2. Are polymorphs predictable from molecular structure?

In the last 20 years or so, there has been enormous progress in the field of crystal structure prediction (CSP)^5^. CSP is a computational technique allowing for the generation of all possible crystal packings of a given compound. A polymorphic landscape is produced whereby hundreds of crystal structures are generated computationally, and their lattice energies calculated. These landscapes are sometimes successful at predicting the most stable low temperature crystal structure, if extremely accurate energy models are used^6^. Predicting which polymorphs might appear experimentally, however, still remains a major challenge. The fact that a crystal structure is generated computationally does not mean it can be obtained experimentally^7^ and indeed making the link between prediction and practical realisation remains an unsolved, yet vital goal simply because while thermodynamic stability can be computed from structural information, rate constants for nucleation and growth cannot yet. However, some progress is being made here. Increasing evidence is appearing for the direct link between nucleation and growth rates with fast growers also being fast nucleators^8^. In this sense the problem may reduce to the computation of growth rates for which the literature is replete with methodologies. Here we mention the simple attachment energy methodology, the mechanistic models of Snyder and Doherty^9^ and most recent work in which surface rugosity is used as a comparative measure of growth rates^10^.

## Experimental preparation of polymorphs

### Q3. Why are some polymorphs so difficult to crystallise?

Nucleation, the origin of a crystal, is perhaps the least well understood step in the area of polymorphism. Kinetics and thermodynamics play a fine game in nucleation and crystal growth of various polymorphs. For example, theophylline form IV, despite being the most thermodynamically stable polymorph, is extremely difficult to crystallise because it is hard to nucleate and grows much slower than the common form II^11^. Ostwald’s rule of stages, with its central tenet that metastable forms will always appear first, has provided a useful experimental guide to the kinetics of solution crystallisation processes^12^. However, recently the rule has been shown to be only a special case of a much bigger experimental landscape, the details of which are currently beyond computation. Gaining a better understanding of the kinetics of crystal nucleation and growth for a wide range of polymorphic systems^13^ is thus a must if we are able to establish better links between crystal forms and the conditions that afford them. Beyond detailed kinetic studies, the community would benefit from abundant and accurate reports of exact crystallisation conditions that lead to new polymorphs. The problem here, however, lies in the fact that often the presence of small amounts of impurities may play an important role. As a consequence, reports of irreproducibility of polymorphic observations or of disappearing polymorphs abound not only across labs but also within one’s own^14^. Another aspect to consider in the crystallisation of polymorphs is the conformational problem whereby polymorphs with high energy conformers have been found to be very difficult to produce^2^. Other polymorphs cannot be nucleated directly from solution but have to be produced via desolvation of intermediate solvates^15^ or through mechanochemistry^16^.

### Q4. Do polymorphs change stability under different conditions?

Most CSP landscapes only illustrate the system state at one unique set of conditions, usually 0 K and no external pressure. Polymorphs, however, are known to change in stability with conditions such as temperature, pressure and even particle size^17^. Therefore, the generation, understanding and prediction of phase diagrams under a number of conditions is an area that deserves careful further consideration within the context of polymorphism. In recent years, a number of approaches have been developed for the computation of crystal free energies^18^ (beyond lattice energies) but these remain computationally expensive. Similarly, exploring polymorph stabilities experimentally with pressure may lead to stability changes between forms and the realisation of new polymorphs^19^. Finally, polymorph stability changes as a function of crystal size have rarely been studied although this has been shown to be important in the context of mechanochemistry^17^ and crystallisation in nanoconfinement^20^.

### Q5. Can the crystallisation of polymorphs be directed by templating?

Many polymorphs can only be obtained by crystallisations in the presence of other compounds either in solution, with solid polymers^21^ or crystallisation within gels^22^. Growing compounds onto isostructural (or mixed) seeds of related analogues has been proven as a way to produce some predicted forms but only in a handful of examples including form V carbamazepine^23^ and ROY^24^. When such templating strategies are possible and how to achieve them remain open questions.

## Characterisation of polymorphs

### Q6. Are we able to detect and determine all polymorphs?

Despite advances in instrumentation over the last 50 years often compounds, especially large flexible pharmaceuticals, are difficult to crystallise as single crystals amenable to X-ray diffraction. In those cases, structure solution from powders or from a combination of methods may be the only possibility. In recent years, there has been some development in electron diffraction techniques which are able to solve complex structures, and even determine absolute configurations, from crystals of just a few hundreds of nm in size^25^. These techniques are still in need of further development so that they may be used more routinely in solid-state labs around the world. Related to this, tiny amounts of other polymorphs may be present in final products but are not detectable with current analytical techniques^26^. Increased sensitivity of analytical methods at the lab scale is therefore required, though this remains an issue of characterisation of materials in general. Finally, it is possible for true polymorphs to have very similar structures to the extent that that they may be overlooked experimentally, having only subtle differences in the X-ray diffraction patterns. Hence expert use of a combination of structural analysis techniques will be important in the future to uncover the true extent of polymorphism^27^.

## Structural basis for physical properties of polymorphs

### Q7. Do crystal dynamics, defects and disorder effect polymorph properties?

The notion of an idealised perfect crystal is only true in theory. Real crystals are full of defects and many also have structural disorder. Beyond our average static view of crystals (averaged positions determined by XRD often at low temperatures), molecules are able to vibrate, librate and in some cases some of their groups are able to rotate (i.e. methyl group rotations or pedal motions). Our view of crystals and polymorphs is too static and to be able to better understand and model disorder or entropic contributions to free energies, we need to move towards a better understanding of crystal dynamics. This may require of further experimental data on high temperature structures and their dynamics as well as dynamic simulations of crystals. Disorder can manifest in many ways. It can be static or dynamic positional disorder of parts of the molecule within the unit cell, as well as more macroscopic phenomena such as mosaicity, twinning or stacking faults possibly also with loss of order at the crystal surfaces^28,29^. Each of these will have a precise effect on the physical properties of ‘real’ crystalline materials yet they remain difficult to characterise experimentally and model computationally even by expert practitioners.

### Q8. Can we predict structure-property relationships?

We need to start establishing links between structure and properties in polymorphs so that we can enable computational prediction^30^. For this, first, properties of polymorphs need to be accurately measured, reported and reviewed. For example, Pudippeddi and Serajuddin compiled the solubility differences of a large set of polymorphs revealing solubility ratios between polymorphs is usually less than 2^31^. In recent years, with the development of nanoindentation there has been some novel work in trying to link crystal structure to mechanical properties of crystals^32^. More such studies would benefit the field tremendously since ultimately, the exploitation of polymorphs will be determined by their physical properties.

## Outlook

In theory, one would want to generate the polymorphic landscape of a compound computationally, link it to crystal properties, retrieve the crystallisation conditions of the desired form and crystallise it. In practice, computationally generated polymorphic landscapes are challenging, structure-property relationships are not yet accurately predictable, we can rarely design crystallisation conditions for the discovery of specific polymorphic forms and crystallisation process design remains a challenging engineering exercise. Whilst we have learnt so much in the last fifty years or so, many fundamental questions remain open for us to solve in the coming decades.
